# Cross-Talk between Oxidative Stress and m^6^A RNA Methylation in Cancer

**DOI:** 10.1155/2021/6545728

**Published:** 2021-08-24

**Authors:** Baishuang Yang, Qiong Chen

**Affiliations:** ^1^Department of Geriatrics, Xiangya Hospital, Central South University, Changsha, China; ^2^National Clinical Research Center for Geriatric Disorders, Xiangya Hospital, Changsha, China

## Abstract

Oxidative stress is a state of imbalance between oxidation and antioxidation. Excessive ROS levels are an important factor in tumor development. Damage stimulation and excessive activation of oncogenes cause elevated ROS production in cancer, accompanied by an increase in the antioxidant capacity to retain redox homeostasis in tumor cells at an increased level. Although moderate concentrations of ROS produced in cancer cells contribute to maintaining cell survival and cancer progression, massive ROS accumulation can exert toxicity, leading to cancer cell death. RNA modification is a posttranscriptional control mechanism that regulates gene expression and RNA metabolism, and m^6^A RNA methylation is the most common type of RNA modification in eukaryotes. m^6^A modifications can modulate cellular ROS levels through different mechanisms. It is worth noting that ROS signaling also plays a regulatory role in m^6^A modifications. In this review, we concluded the effects of m^6^A modification and oxidative stress on tumor biological functions. In particular, we discuss the interplay between oxidative stress and m^6^A modifications.

## 1. Introduction

Reactive oxygen species (ROS) are byproducts of the respiratory chain, which act as important signal transduction molecules in cells [1]. The production of ROS is regulated by a variety of intracellular and extracellular stimuli. These oxygen-based molecules contain unpaired electrons, and their instability can lead to the irreversible inactivation of intracellular targets such as proteins, nucleic acids, and lipids [2]. Under increased ROS production, cells protect themselves from ROS damage by producing enzymatic antioxidants (e.g., superoxide dismutase (SOD), catalase (CAT), and glutathione peroxidase (GPX)) and nonenzymatic antioxidants (e.g., glutathione and thioredoxin) [3]. An imbalance in the relative abundance of ROS and antioxidants can lead to profound pathophysiological consequences. Oxidative stress is defined as a relative excess of ROS, which is closely associated with aging-related diseases, such as neurodegenerative disorders [4], cardiovascular diseases [5, 6], and normal senescence [7, 8], and with many other diseases, including cancer. Many oncogenes can affect ROS production in a direct or indirect manner; thus, cancer cells usually show elevated levels of ROS. To adapt to the relatively high levels of ROS and maintain survival and proliferative activity, the antioxidation capability of cancer cells is increased to neutralize the cytotoxicity caused by excessive ROS [9]. Significantly, oxidative stress impacts tumor development in a concentration-dependent manner. Elevated ROS levels generally participate in promoting cancer, but excessive ROS levels generate toxicity to cancer cells [10].

N^6^-methyladenosine (m^6^A) is the most common type of internal RNA modification in eukaryotes [11]. With the development of high-throughput sequencing, it has been found that m^6^A modifications are mainly enriched in 5′-untranslated regions (5′-UTRs), stop codons, and 3′-untranslated regions (3′-UTRs) and are located in specific RRACH (R=G/A, A=m^6^A, H=U/A/C) motifs in RNA [12, 13]. The location and distribution of m^6^A modifications at the transcriptome level are gradually being revealed. m^6^A marks are widely distributed in 6990 mRNAs and 250 noncoding RNAs that regulate maturation, transcription, translation, and metabolism and are involved in the modulation of various pathological and physiological activities. Changes in m^6^A levels have profound effects on numerous cellular processes, including autophagy [14], the DNA damage response [15], oxidative stress [16], and tumorigenesis [17]. m^6^A modifications and oxidative stress play complex and contradictory roles in tumorigenesis and development. Surprisingly, m^6^A modifications show widespread, close interrelationships with oxidative stress. m^6^A modifications affect oxidative stress-related genes' expression which have different effects on oxidative stress, thus affecting the generation and development of cancer [18–20]. On the other side, the expression and activity of m^6^A enzymes and m^6^A levels can be dynamically regulated by ROS [21, 22]. A systematic and in-depth understanding of m^6^A and oxidative stress in tumor formation and progression is thus of great significance for the diagnosis and treatment of cancer. Therefore, we describe the way in which oxidative stress and m^6^A modification influence biological functions in cancer and discuss the cross-talk between them in this review.

## 2. N^6^-Methyladenosine in Cancer

### 2.1. Writers

m^6^A regulatory factors can be classified into three categories: “writers”, “erasers” and “readers”. “Writers” are a set of proteins that participate in the formation of the m^6^A methyltransferase complex (MTC) and catalyze m^6^A modification by using S-adenosylmethionine as a methyl donor. The complex consists of methyltransferase-like 3 (METTL3), METTL14 and their cofactors WT1-associated protein (WTAP), Vir-like m^6^A methyltransferase associated (VIRMA), zinc finger CCCH-type containing 13 (ZC3H13), and RNA-binding motif protein 15/15B (RBM15/15B) [23–27]. METTL3 plays a major catalytic role in the MCT. Knockdown of METTL3 leads to almost complete loss of m^6^A modification activity [28]. METTL14 interacts with METTL14 to form a METTL3-METTL14 heterodimer through an extensive hydrogen-bonding network. It acts as an RNA-binding platform in m^6^A methylation and enhances the catalytic activity of METTL3 through allosteric activation. However, METTL14 itself cannot directly promote methyl transfer [29]. WTAP interacts with the METTL3-METTL14 heterodimer to stabilize MTC and promote the localization of the core complex to nuclear speckles. Knockdown targeting WTAP caused a significant reduction in the RNA-binding capability of METTL3, suggesting that WTAP plays a critical role in RNA modification by regulating the recruitment of MTC to RNA targets [25]. Other evidence has shown that RBM15/15B[27], VIRMA [30], and ZC3H13[31] may be components of the MTC and function as regulators to bind and recruit the complex to affect the stability and location of the MCT and thereby regulate the methylation modification process. METTL16 is a newly discovered m^6^A methyltransferase that can catalyze the m^6^A modification of U6 spliceosomal small nuclear RNA (snRNA) and participate in cotranscriptional and posttranscriptional splicing [32].

### 2.2. Erasers

The reversibility of the m^6^A modification relies on demethylases, also known as “erasers”. Fat mass and obesity-associated protein (FTO) and alkB homolog 5 (ALKBH5) are independent m^6^A demethylases that perform demethylation functions and require the involvement of the cofactor Fe(2+) and *α*-ketoglutarate (*α*-KG) or 2-oxoglutarate (2-OG). FTO partially colocalizes with nuclear speckles. The distribution of FTO in cells determines its effect on different RNA substrates [33]. FTO knockdown can increase the amount of m^6^A in mRNA, while FTO overexpression results in a decrease in m^6^A modifications [34]. ALKBH5 is another RNA demethylase that is expressed in most tissues, is mainly located in the nucleus, and can remove m^6^A residues from mRNA in vitro and in vivo. The deletion of the ALKBH5 gene leads to a remarkable reduction in mRNA levels in the cytoplasm, suggesting that its demethylation activity notably affects mRNA export as well as RNA metabolism [35].

### 2.3. Readers

“Readers” are a set of m^6^A-binding proteins that specifically recognize and mediate the biological functions of m^6^A modified RNA. Proteins with conserved m^6^A-binding domains, including YTHDC1-2 and YTHDF1-3, are the main m^6^A readers that belong to the YT521-B homology (YTH) domain family. YTHDC1 is the core member of the YTH domain family and can selectively recruit and modulate pre-mRNA splicing factors and regulate RNA alternative splicing after recognizing m^6^A in the nucleus [36]. YTHDC2 is an m^6^A-binding protein that contributes to enhancing the translation efficiency of target RNAs and simultaneously reduces their abundance [37]. YTHDF1 interacts with eukaryotic initiation factors (eIFs) and ribosomes to accelerate the translation of m^6^A-modified mRNAs [38]. YTHDF2 accelerates the degradation of m^6^A-modified transcripts by selectively binding with them and directing the complex to cellular RNA decay sites [39]. YTHDF3 not only works in conjunction with YTHDF1 to expedite mRNA translation but also participants in the process of YTHDF2-mediated mRNA decay [40, 41].

Heterogeneous nuclear ribonucleoproteins (HNRNPs), eIFs, and other special proteins also act as m^6^A readers. The HNRNP family is a series of nuclear RNA-binding proteins (including HNRNPC, HNRNPG, and HNRNPA2B1) that influence mRNA precursor processing [42]. In addition to participating in the maturation of mRNA, HNRNPA2B1 and METTL3 have also been proven to affect the beginning of microRNAs (miRNAs) biogenesis, primary miRNA processing, and alternative splicing, in the early stage of microRNA (miRNA) biogenesis by interacting with the microprocessors complex DiGeorge syndrome critical region 8 (DGCR8, [42–44]. eIF3 initiates translation in a cap-independent manner by directly binding to the m^6^A site of the mRNA 5′-UTR and recruiting the 43S complex [45]. Studies have revealed that insulin-like growth factor 2 mRNA-binding proteins (IGF2BPs) recognize the consensus m^6^A site GG (m^6^A) C and target mRNA transcripts to maintain their stability and thus increases the levels of its stored target mRNAs [46]. Furthermore, a novel m^6^A reader identified in recent research, proline-rich coiled-coil 2 A (Prrc2a), binds to a GG (m^6^A) CU motif of the target coding sequence and stabilizes its target mRNA in an m^6^A-dependent manner [47]. The FMRP Translational Regulator 1 (FMR1) and Leucine-Rich Pentatricopeptide Repeat-Containing (LRPPRC) proteins can also read m^6^A modifications and affect RNA behavior [48, 49]. Recent studies have reported that FMR1 directly interacts with YTHDF1 to inhibit the translation of target transcripts [50].

### 2.4. Role of m^6^A in Cancer

The dynamic reversibility of m^6^A affects gene expression and numerous cellular processes. A recent wave of studies has shown that m^6^A modification regulates RNA maturation, transcription, translation, and metabolism, which are involved in the modulation of various physiological activities. Therefore, imbalances in m^6^A levels lead to a variety of diseases, especially cancers. The abnormal modification and expression of m^6^A regulatory proteins can be detected in multiple tumor types and modulates the expression of tumor-related genes [51]. Changes in m^6^A levels may profoundly affect the processes of tumor growth, progression, and metastasis, including proliferation signaling [51], angiogenesis [52, 53], cell development and differentiation [54], cellular metabolic reprogramming [52, 55, 56], immune responses and evasion [57, 58], and inflammation [51, 59]. Due to the extensive and complex functions of m^6^A methylation, it plays dual roles in cancer: a high m^6^A level may lead to oncogenesis, but the deletion of m^6^A methylation modifications may lead to the progression of other tumors (Table 1).

## 3. Overview of Oxidative Stress in Cancer

ROS are chemical species that form highly active radicals on the unpaired electrons of oxygen. They are generally considered to include reactive oxygen compounds such as superoxide (O_2_^•−^) and hydroxyl (HO•) free radicals and nonradical molecules such as hydrogen peroxide (H_2_O_2_), singlet oxygen (^1^O_2_), and ozone (O_3_) [60]. The most important source of ROS is the mitochondria. Approximately 2% of oxygen can receive single or double electrons from the middle portion of the electron transport chain (ETC) and be partially reduced to O_2_^•−^/H_2_O_2_ [61, 62]. NADPH oxidases (NOXs) are other sources of intracellular oxidants that are located on the cell membrane, nuclear membrane, or endoplasmic reticulum membrane. NOXs can transfer electrons from reduced NADPH to catalytic superoxide and other downstream ROS [63, 64]. In addition to NOXs and the ETC, ROS are produced by other enzymes in organelles such as the endoplasmic reticulum (ER) and peroxisomes, including xanthine oxidase (XO), endothelial nitric oxide synthase (eNOS), lipoxygenase (LOX), cyclooxygenase (COX), and cytochrome P450 reductase (POR) [65–67].

ROS are gradually coming to be considered important signal transduction molecules or regulators in biological systems, rather than only byproducts of metabolism [1]. Different ROS levels have different biological effects. Under physiological conditions, small amounts of ROS are produced in cells, which involve in cell proliferation and differentiation by activating stress-responsive survival signals, and their toxicity is easily offset by the antioxidant defense system. However, excessive ROS levels can exert toxicity, leading to cell dysfunction and even death. Consequently, the maintenance of normal physiological function depends on the balance between oxidants and antioxidants. The three-layer antioxidant defense system maintains the redox homeostasis of cells. Uric acid, glutathione (GSH), and vitamins C and E belong to small-molecule antioxidants which can scavenge ROS directly. The antioxidant enzymes that play roles in intermediate defense include SOD [68], CAT [69], GPX [70], thioredoxin (Trx) [71], and peroxiredoxin (Prx) [72], which catalyze the transformation of ROS into less cytotoxic products. Enzymes that repair or remove damaged biomolecules, such as 8-oxoguanine glycosylase (OGG1), apurinic/apyrimidinic endonuclease (APE1), and DNA polymerase, have generally been regarded as the last line of defense in the repair of oxidative damage [73–75].

The production of ROS in malignant tumors increases and contributes to maintaining the cancer phenotype. Similar to normal cells, the intracellular sources of ROS produced by cancer cells contain mitochondrial ETC, NOX, ER, and LOX [10]. Damage stimulation and the excessive activation of oncogenes lead to elevated ROS production in cancer. Abnormal activation of Kirsten rat sarcoma viral oncogene homolog (KRAS) and the amplification of MYC protooncogene (MYC) in cancer enhance the catabolism of glutamine as the carbon source of the tricarboxylic acid cycle, increasing the ROS generation by ETC [76, 77]; mutant KRAS can also increasing mitochondrial ROS via decreasing the stabilization of electron transport and leading to the leakage of electrons [78]; B-cell lymphoma 2 (Bcl-2) is overexpressed in a variety of tumors which can affect the activity of ETC by interacting with cytochrome C oxidase [79]. Oncogenes also mediate ROS production by regulating NOXs expression. Signal transducer and activator of transcription 3 (STAT3) increases NOX4 expression [80]; mutant KRAS activates NOX1 and downregulates antioxidant enzymes, including SOD2, catalase, and Prxs [81, 82]; and Ras-related C3 botulinum toxin subunit 1 (Rac1) can stimulate the production of mitochondrial superoxide and participate in the assembly and activation of NOX1 and NOX3 [83–86]. Tumor suppressor p53 and forkhead box O (FOXO) family transcription factors can prevent oxidative stress by inducing the expression of antioxidant genes. The inactivation of these transcription factors in tumors may increase ROS production [9, 87]. In addition, transforming growth factor *β* (TGF-*β*) stimulates fibroblasts to induce NOX4 upregulation and elevate ROS production during tumor-related matrix remodeling [88].

One of the most important reasons for the enhanced antioxidant capacity in cancer is the regulation of the redox homeostasis of cancer cells by nuclear factor erythroid-2-related factor 2 (Nrf2) [89]. In the process of cancer development, Nrf2 expression can be increased by activating oncogenes (such as KRAS) or environmental signals (such as hypoxia) [90, 91], the loss of the negative regulator Kelch-like ECH-associated protein 1 (Keap1) directly activates Nrf2 [89], and elevated levels of ROS prevent the proteasome-mediated degradation of Nrf2. Nrf2 is a transcription factor whose increased expression and activity initiate the transcription of various antioxidant genes [92]. In addition, NADPH metabolism enzymes and NAD (P)H: quinone oxidoreductase 1 (NQO1), which inhibits the formation of free radicals, are regulated by Nrf2 [93, 94]. Therefore, Nrf2 is regarded as a stress reliever that maintains a high but balanced redox state in tumors and supports cancer cell survival. Another protein that restricts ROS is TP53-induced glycolysis regulatory phosphatase (TIGAR), a protein with bisphosphatase activity that is involved in activating the oxidized pentose phosphate pathway (PPP), thereby increasing the production of NADPH for antioxidant defense [95, 96].

## 4. Effect of Oxidative Stress on Cancer

**Figure 1:**
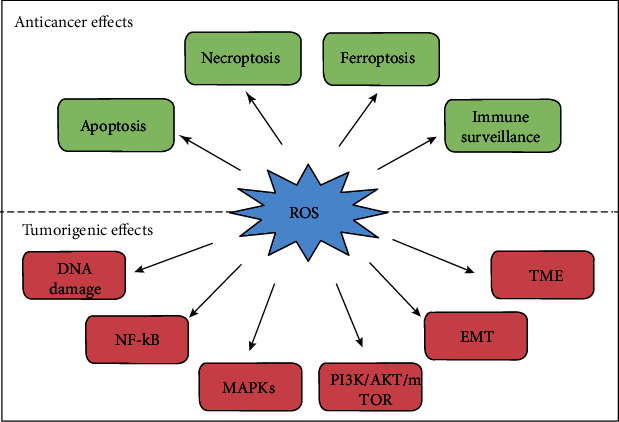
The dual effects of oxidative stress in cancer progression. ROS play an anticancer role by promoting apoptosis, necrosis, and ferroptosis of cancer cells and enhancing the immune surveillance ability of immune cells. Conversely, ROS promoting cancer progression by inducing DNA damage and genomic alterations, activating cell proliferation-related pathway (NF-B, MAPKs, and PI3K/AKT/mTOR), accelerating EMT, and altering the tumor microenvironment for cancer invasion and metastasis.

Previous studies have evaluated the levels and production of ROS in cancer cells under different conditions, clarifying the relationship between ROS and tumor growth [97]. Excessive ROS play an antitumor role by inducing DNA damage, cell death, and aging, whereas elevated levels of oxidants and antioxidants support the proliferation and survival of cancer cells (Figure 1) [98, 99]. In different types of tumors, oxidative stress mediates anticarcinogenic effects or cancer-promoting effects through different mechanisms (Table 2).

### 4.1. Tumorigenic Effect of Oxidative Stress

As we mentioned earlier, more elevated ROS levels showed in cancer cells than normal cells, and the antioxidant capacity is enhanced to counteract the toxicity of excessive ROS. An abnormal redox balance may play a carcinogenic role through different mechanisms.

#### 4.1.1. Oxidative Stress Induces DNA Damage and Genomic Alterations

Carcinogenic stimulation, increased metabolic activity, and mitochondrial dysfunction lead to increased ROS levels in cancer cells [100]. As early as the 1990s, it was recognized that ROS-mediated DNA damage can induce gene mutation [101]. Excessive ROS can attack various components of DNA, resulting in DNA intrastrand adducts, oxidized bases, strand breaks, and DNA-protein crosslinks [102, 103]. Notably, mitochondrial DNA is more susceptible to damage than nuclear DNA because it is located closer to the site where ROS are produced. ROS-mediated mitochondrial DNA damage may lead to respiratory chain dysfunction, further amplifying oxidative stress and thereby destroying genome functions, inducing genome instability, and increasing the risk of mutations [100]. 8-Oxo-7,8-dihydro-2′-deoxyguanosine (8-oxo-dG) is an oxidative adduct produced by ROS-related DNA damage and is a common mutagenic structure in DNA that can be repaired mainly through OGG1-mediated base excision [104]. ROS affect DNA repair by inhibiting OGG1 activity [105]. In summary, ROS can alter the balance of DNA damage repair functions in cancer cells, causing ROS-mediated DNA damage to far exceed the repair ability, which leads to the accumulation of multiple mutations and, ultimately, carcinogenic mutations and tumorigenic transformation.

ROS can be involved in tumorigenesis by regulating oncogenes, tumor suppressor genes, and DNA repair genes [104, 106]. Elevated ROS promote the occurrence of cancer by inducing oxidation and base pair substitution mutations of these tumor-related genes [107]. The members of the rat sarcoma (RAS) viral oncogene family, which include HRAS, KRAS, and NRAS [108], are the most commonly mutated oncogenes in human cancers [109]. Activated KRAS has been proven to upregulate NOX and increase superoxide production and consequent malignant transformation [110, 111]. In addition, mitochondrial ROS are essential for KRAS^G12D^-induced tumorigenesis [76]. Increased ROS conversely promote the expression of KRAS and Nrf2 under oxidative stress, and the ectopic expression of KRAS^G12D^ or KRAS^G12V^ stimulates Nrf2[112], supporting KRAS^G12D^-driven tumor development [90]. The tumor suppressor gene tumor protein p53 (TP53) inhibits tumorigenesis by inducing cell growth arrest or apoptosis. ROS can induce G-to-T mutations in TP53 [113]. p53 protects the genome from ROS oxidation by enhancing DNA repair and upregulating the expression of antioxidant genes. Therefore, p53 loss-of-function mutations lead to further increases in intracellular ROS levels, cause excessive DNA oxidation, increase mutations and karyotype instability, and promote tumor development [114].

#### 4.1.2. Oxidative Stress Promotes Tumor Cell Proliferation

Many classical pathways involved in the ROS-mediated proliferation of cancer cells. Nuclear factor-*κ*B (NF-*κ*B) (nuclear factor of kappa light polypeptide gene enhancer in B cells) plays a key role in multiple cellular processes including immune and inflammatory responses and cell proliferation and differentiation [115, 116]. The typical NF-*κ*B pathway can be activated in response of oxidative stress [115]. ROS induce the phosphorylation of I*κ*B kinase *α* (I*κ*K*α*), which results in the ubiquitination and degradation of NF-*κ*B inhibitor *α* (I*κ*B*α*), thereby promoting translocation of NF-*κ*B heterodimers to the nucleus and thus increasing the transcription of downstream target genes of the NF-*κ*B pathway [117]. The abnormal activation of NF-*κ*B promotes the growth, proliferation, and angiogenesis of a variety of cancers by upregulating the expression of antiapoptotic genes, cyclins, protooncogenes, matrix metalloproteinases (MMPs), and cell adhesion genes [118]. NF-*κ*B is also conducive to conversion from oxidative phosphorylation to glycolysis in cancer cells [119] and promotes the survival and proliferation of cancer cells by regulating cellular components in the tumor microenvironment [120, 121]. In hepatocellular carcinoma cells, treatment with H_2_O_2_ and N-acetylcysteine (NAC, a kind of ROS scavenger) was shown to alter intracellular ROS levels, and the results showed that the activity of NF-*κ*B increased after exposure to H_2_O_2,_ while the opposite result was obtained after NAC treatment [122]. In internal stem cells (ISCs), Rac1-driven ROS and NF-*κ*B signaling mediate progenitor cell proliferation and transformation [123].

ROS participate in PI3K/AKT/mTOR and MAPK/ERK signal-mediated activation of growth factors. Phosphatase and tensin homolog (PTEN), a lipid phosphatase that is sensitive to redox reactions, is one of the most frequently deleted and mutated antioncogenes in human cancers. PTEN can coordinate cell proliferation, growth, and survival by negatively regulating the PI3K/AKT/mTOR signaling pathway [124, 125]. H_2_O_2_ treatment results in the time- and concentration-dependent inactivation of purified PTEN in vitro [126]. Other studies have shown that H_2_O_2_ can oxidize cysteine residues of PTEN, resulting in its temporary inactivation, and can induce the activation of downstream protein kinase B (AKT) [127, 128]. Similarly, H_2_O_2_ catalyzes the reversible oxidation of protein tyrosine phosphatase 1B (PTP1B) [129]. Like PTEN, PTP1B is a negative regulator of phosphoinositide 3-kinase (PI3K) and AKT. ROS-mediated oxidative inactivation of PTEN and/or PTP1B can induce the overactivation of the PI3K/AKT/mTOR pathway, which is characteristic of malignant tumors [130, 131]. Endogenous antioxidants (e.g., Trxs and Prxs) regulate the intracellular redox state. An increase in Trx1 levels can cause Trx1 to bind to PTEN in a redox-dependent manner and inhibit its lipid phosphatase activity, leading to an increase in AKT activation in cells and thus promoting tumorigenesis [132]. However, PTEN can be reactive by thioredoxin-interacting protein- (TXNIP-) mediated pathways. [133]. Prxs regulate intracellular H_2_O_2_ levels by catalyzing hydrogen peroxide reduction [134]. Prx1 interacts with PTEN to protect and promote the antitumor function of PTEN under mild oxidative stress. However, Prx1 separates from PTEN and irreversibly loses its peroxidase activity under high concentrations of H_2_O_2_ (500 *μ*M)[135].

MAPKs regulate proliferation, differentiation and apoptosis, and other cellular activities related to tumors. MAPKs mainly include four subgroups: c-Jun NH2 terminal kinase (JNK), extracellular signal-regulated kinase (ERK), big MAP kinase 1 (BMK1/ERK5), and p38 kinase (p38) [136]. Apoptosis signal-regulating kinase 1 (ASK1) is a kind of MAPK kinase kinase kinase (MAPKKK) that can activate MAPK cascades. The reduced form of the redox regulatory protein Trx binds to ASK1 to inhibit its activity, and this interaction can be reversed, thereby restoring ASK1 kinase activity, when ROS accumulation or a lack of antioxidants induces Trx oxidation [137]. ROS can also activate MAPKs directly by inhibiting MAPK phosphatases. The inhibition of JNK-inactivating phosphatases occurs through the reversible oxidation of cysteine residues to sulfonic acid by ROS, thereby maintaining the activation of JNK [138]. In addition, the oxidation of p53 cysteine residues has been shown to affect the ability of p53 to bind DNA, affecting downstream gene expression. Therefore, ROS may disrupt the cell cycle regulation function of p53, leading to uncontrolled cell proliferation [87]. In general, ROS can affect the abovementioned signal transduction pathways and stimulate cell proliferation.

#### 4.1.3. Oxidative Stress Accelerates Tumor Invasion and Metastasis

The dissemination and colonization of primary tumor cells to invade distant organs are known as tumor metastasis. Epithelial-to-mesenchymal transition (EMT) is an early change that occurs during tumor metastasis. EMT is characterized by cytoskeletal reorganization, loss of epithelial morphology and markers (E-cadherin, desmoplakin, Muc-1, cytokeratin-18, *γ*-catenin, etc.), and increased expression of mesenchymal markers (N-cadherin, vimentin, fibronectin, *α*-smooth muscle actin (SMA), etc.) and MMPs [139–141]. ROS can participate in tumor metastasis by regulating EMT. TGF-*β*1 is considered to be an important inducer of EMT that activates NF-*κ*B through ROS-dependent pathways, upregulates urokinase-type plasminogen activator (uPA) and MMP9, and thus promotes cell migration and invasion [142]. The deletion of TGF-*β*-activated kinase 1 (TAK1) enhances and accelerates EMT by negatively regulating RhoA through Rac-induced ROS [143]. Due to the uncontrolled division of cancer cells, there is an insufficient nutrition and oxygen supply in the tumor microenvironment; thus, cancer cells are in a state of hypoxia. Hypoxia-inducible factor (HIF) plays a key role in EMT in tumor cells. HIF targets the EMT promoter snail and promotes hypoxia-induced EMT in different tumor types [144–146]. Complex III of ECT is essential for stabilizing HIF-1*α* under hypoxic conditions by increasing ROS production [147]. ROS can also enhance the transcription of HIF-1*α* by phosphorylating ERK and PI3K/AKT during hypoxia [148, 149]. Inflammatory mediators also facilitate ROS-mediated alteration of HIF-1*α* transcription and translation. GSH depletion or exogenous ROS enhance the production of inflammatory mediators such as tumor necrosis factor-*α* (TNF-*α*) and interleukin-1*β* (IL-1*β*) [150], which induce HIF-1*α* transcription and continuous protein synthesis at room temperature [151]. Furthermore, mild oxidative stress increases the transcriptional activity of HIF-1*α* by regulating the stability and nuclear localization of Sentrin/SUMO-specific proteases (SENPs) [152]. Prolyl hydroxylase (PHD) family members play crucial roles in stabilizing HIF by acting as oxygen sensors. The prolyl residues of PHD are hydroxylated under aerobic conditions and bind to the tumor suppressor protein Von Hippel Lindau (pVHL), which is a component of a ubiquitin ligase complex. Therefore, proteasomal degradation of HIF-1*α* occurs in the presence of sufficient oxygen [153]. Exogenous H_2_O_2_ treatment can stabilize the HIF-1*α* protein by inhibiting the prolyl hydroxylation of PHD under normoxic conditions [147, 154], which can be reversed by vitamin C [155]. The loss of the antioxidant protein TIGAR is related to enhanced ROS production, which can promote the occurrence of EMT in pancreatic cancer cells by activating ERK. These phenotypes can be reversed by ROS limitation signaling [156].

The development of the tumor microenvironment (TME) maintains an appropriate environment for tumor growth, invasion, and metastasis. Tumor-associated macrophages (TAMs) are critical regulators of tumorigenesis that drive aggressive cancer phenotypes [157]. Oxidative stress affects the differentiation of macrophages. A study indicated that ROS promote the differentiation of monocytes by activating ERK, while ROS inhibitors reduce the differentiation of M2-like tumor-promoting phenotypes [158]. ROS can also induce cancer cells to release the KRAS^G12D^ protein, which can be taken up by macrophages, leading to tumor-promoting transformation, thereby promoting the growth of pancreatic cancer [159]. Cancer-associated fibroblasts (CAFs), transformed from normal fibroblasts or fibroblast progenitor cells, can promote tumor proliferation, EMT, angiogenesis, tumor invasion, and immunosuppression by helping remodel the extracellular matrix (ECM) [160]. TGF-*β* is considered the main mediator of CAF activation [161]. The transcription factor JunD regulates antioxidant genes, and chronic oxidative stress caused by its deletion increases the migratory properties of stromal fibroblasts by inducing the accumulation of HIF-1*α* and CXC chemokine ligand 12 (CXCL12), thereby promoting tumor spreading [162]. The premetastatic niche determines whether circulating tumor cells can colonize and survive in distant sites, which is the final stage of successful tumor metastasis [163]. There is evidence that ROS play supporting roles in the tumor environment colonized by cancer cells. ROS inhibit the activity of cytotoxic CD8^+^ T cells, thereby promoting the survival of disseminated cancer cells at a secondary tumor site [164]. Lactic acid production lowers the pH value of the TME in colorectal cancer, leading to mitochondrial ROS accumulation and the apoptosis of liver NK cells, thereby promoting the occurrence of colorectal cancer liver metastasis [165].

### 4.2. Anticancer Effect of Oxidative Stress

#### 4.2.1. Oxidative Stress Activates Apoptosis Pathways

Apoptosis is a kind of programmed cell death whose execution depends on apoptotic effector caspases [166]. The endogenous apoptotic pathway, also known as the mitochondrial apoptotic cascade, is regulated by mitochondrial Bcl-2 proteins. The Bcl-2 family increases the permeability of mitochondria, allowing the release of the proapoptotic factor cytochrome C, which then activates the caspase-9 signaling cascade and induces apoptosis [167, 168]. These are the key steps in the endogenous cell apoptosis process [169]. ROS stimulation facilitates cytochrome C release and induces the downstream apoptotic cascade by depleting the mitochondrial membrane potential, changing the permeability of the mitochondrial membrane, and oxidizing cardiolipin, leading to cytochrome C dissociation [170]. The overexpression of the antioxidant enzyme glutaredoxin 2 (GRX2) in HeLa cells can reduce cytochrome C dissociation and resist caspases [171]. ROS also increase mitochondrial outer membrane permeability by regulating the Bcl-2 family [172, 173]. In addition, ROS activate caspase-9 directly by increasing the interaction between oxidative-modified caspase-9 and apoptotic protease activating factor 1 (APAF-1), which in turn promotes caspase-9 activation and apoptosis [174].

The exogenous apoptotic pathway, also known as the death receptor pathway, is initiated by extracellular TNF superfamily members, which act as death ligands that bind to related cell surface death receptors and activate receptor clustering. The activated receptors recruit adaptor proteins, including Fas-associated via death domain (FADD) and TNFRSF1A associated via death domain (TRADD), and procaspase-8 forms a death-inducing signaling complex (DISC), which then induces apoptosis [175]. The cytosolic protein cellular FLICE-inhibitory protein (c-FLIP) is a main antiapoptotic regulator that inhibits DISC generation by inhibiting caspase 8 recruitment [176]. ROS activate the exogenous apoptotic signal by inducing the ubiquitination and degradation of c-FLIP. The pretreatment of prostate cancer cells with active oxygen scavengers can decrease the ROS-induced degradation of c-FLIP protein [177].

#### 4.2.2. ROS Promote Tumor Cell Necroptosis

Necroptosis is a caspases-independent cell death form which mainly mediated by death receptors and their corresponding ligands [178, 179]. The death signal induces the activation of receptor-interacting proteins (RIPs) 1 and 3, which in turn phosphorylate mixed lineage kinase domain-like (MLKL), a specific protein driving cell necrosis. Phosphorylated MLKL (p-MLKL) is recruited into necrosomes and mediates the destruction of cell and organelle membrane integrity, resulting in the leakage of intracellular components and cell death [180–182]. An increasing number of studies have confirmed the role of necroptosis in mediating tumor death and limiting tumor metastasis [183–186]. ROS promote the autophosphorylation of RIP1 via the oxidative modification of RIP1 Cys-257, Cys-268, and Cys-586 residues and then recruit RIP3 to form necrosomes [187]. Mn (III) tetrakis (4-benzoic acid) porphyrin (MnTBAP) removes mitochondrial superoxide, which can decrease ROS production and hinder RIP1 and RIP3 expression [188]. The removal of ROS by the antioxidant butyl hydroxyanisole (BHA) can significantly reduce the necroptosis of mouse fibrosarcoma cells [189]. In addition to the production of mitochondrial ROS, NOX1-induced oxidative stress has been shown to trigger necroptosis [190]. In summary, these results indicate that ROS promote tumor cell necroptosis by regulating the assembly of necrosomes and affecting the expression and activation of RIP1 as well as RIP3.

#### 4.2.3. ROS Trigger Ferroptosis in Cancer

Ferroptosis is a type of cell death relying on iron and ROS. Ferroptosis can be distinguished from apoptosis, necrosis, and autophagy by morphology, biology, and genetics. Free redox-active iron in cells generates ROS through Fenton and/or increased lipoxygenase activity, causing the accumulation of peroxidated polyunsaturated fatty acids (PUFAs) and, thus, leading to cell death [191, 192]. The tumor suppressor gene p53 makes cells sensitive to ferroptosis by inhibiting the expression of solute carrier family 7 member 11 (SLC7A11). ROS treatment can downregulate the expression of SLC7A11 and maintain ferroptosis in p53 mutant inactivated cells, suggesting that ROS play a key role in ferroptosis [193]. Multiple studies have shown that ROS-triggered ferroptosis strongly inhibits various cancers, including colorectal cancer [194], nasopharyngeal cancer [195], melanoma [196], pancreatic tumors [197], and breast cancer [198].

#### 4.2.4. Oxidative Stress Affects Immune Cells in the TME

The TME is a complex and dynamic environment. Earlier, we introduced the cancer promotion effect of ROS via the induction of malignant cell transformation and ECM remodeling in the TME. In contrast, T cells and natural killer (NK) cells participate in cancer immune surveillance [199, 200]. ROS act as signal mediators to activate T cells [201]. In mice with reduced mitochondrial ROS production, the antigen-specific expansion of T cells cannot be induced, indicating that mitochondrial ROS are key components in the activation of T cells [202]. Hydrogen peroxide influences lymphocyte activation by modulating negative regulatory phosphatases, and it plays an important role in initiating and amplifying signals at antigen receptors by acting as a second messenger [203]. Neutrophils and macrophages also exert tumor-killing effects through ROS [204], in which they release ROS, contributing to tumor killing [205, 206].

## 5. The Interplay between m^6^A Modification and Oxidative Stress

m^6^A methylation and oxidative stress are widely involved in the regulation of tumorigenesis and development. There is a complex relationship between the oxidative stress-related and m^6^A regulating signal pathways. m^6^A methylation of specific RNAs triggers or inhibits oxidative stress has been observed in cancers (Table 3). It is worth noting that ROS can act as an intracellular signal to affect the epigenetic modification of RNA (Table 4). On the one hand, m^6^A affects the survival and invasion of cancer cells by regulating oxidative stress; On the other hand, oxidative stress signals influence the overall and local levels of m^6^A, which may be an adaptive response of cancer cells to environmental changes and external stimuli. Some antioxidants exert regulatory effects on m^6^A modification have been confirmed to play an anticancer role in multiple cancer, while m^6^A-related signals may become a potential predictive or therapeutic marker in cancer for their regulatory role in redox homeostasis. Therefore, to discuss the crosslink between oxidative stress and m^6^A is helpful to understand the mechanism of tumorigenesis and provide the basis for the discovery of novel targets for tumor therapy.

### 5.1. Oxidative Stress Regulates m^6^A RNA Methylation

**Figure 2:**
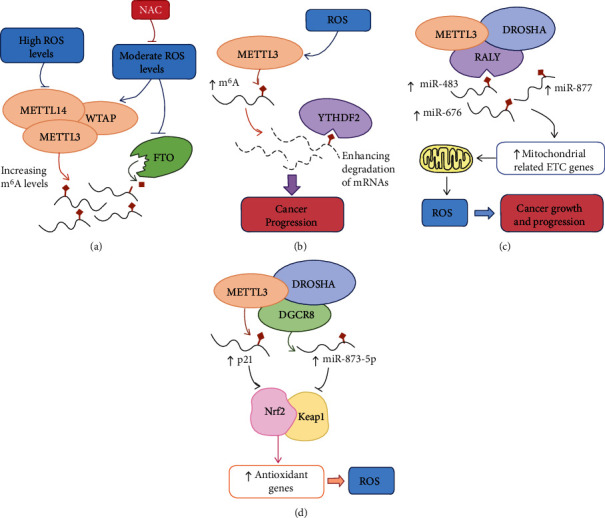
The interplay between m^6^A and oxidative stress. (a) ROS affect m^6^A modification in a dose-dependent manner. (b) ROS influence m^6^A-mediated RNA stability, which is involved in tumor progression. (c) m^6^A promotes tumor growth through mitochondrial function regulation and ROS production. (d) m^6^A regulates the antioxidant response through Nrf2/Keap1 signaling.

In a study that used high-performance liquid chromatography tandem mass spectrometry to identify and quantify m^6^A modifications in highly purified yeast mRNA samples, researchers found that m^6^A-modified mRNA levels changed under oxidative stress [207]. Subsequent studies explored the effect of ROS on m^6^A modification levels in human keratinocytes. The treatment of human keratinous HaCaT cells with the environmental carcinogen arsenite can upregulate methyltransferase (METTL3/METTL14/WTAP) levels, increase the m^6^A modifications mediated by these enzymes, and inactivate the demethylase FTO, which protects cells from oxidative stress and promotes survival. NAC inhibits the increase in methyltransferase and m^6^A levels in human keratinocytes exposed to arsenite. In contrast, after high-dose arsenite treatment, increased oxidative stress induces the downregulation of m^6^A levels, showing an inhibitory effect on HaCaT cell viability (Figure 2(a)) [22, 208]. Oxidative damage induced by CdSO_4_ causes a significant reduction in m^6^A modifications in pancreatic *β*-cells. FTO and METTL3 mRNA levels also decrease in a concentration-dependent manner after CdSO_4_ treatment [209]. These results suggest that different degrees of oxidative stress may have different effects on the m^6^A modification.

Hypoxia is the inducer of ROS production [210]. There are evidences show that ALKBH5 reducing the overall m^6^A level in response of hypoxia [211]. Expression of ALKBH5 can be promoted by HIF-1*α* and HIF-2*α* in a hypoxia condition. Elevated levels of ALKBH5 demethylate the transcripts of pluripotency-related gene Nanog homeobox (NANOG) thereby increasing its expression, and inducing breast cancer stem cell phenotype [212]. In addition, the activity of the demethylase ALKBH5 and the overall m^6^A methylation level is directly regulated by ROS. ROS can induce the posttranslational modification of ALKBH5 by activating the ERK/JNK signaling pathway and thus inhibit its activity, which helps to increase mRNA m^6^A levels and maintain the genomic integrity of cells [213].

Similarly, oxidative stress exerts different effects on m^6^A readers. The deletion of Bmal1 increases ROS production in hepatic cells, resulting in an increase in METTL3-mediated m^6^A mRNA methylation, particularly that of nuclear receptor peroxisome proliferator-activator *α* (PPaR*α*). YTHDF2 binds to PPaR*α* to mediate its mRNA stability to alter hepatic lipid metabolism [214]. YTHDF1 is considered to be a hypoxia-adaptive gene that is highly expressed in various cancers, including NSCLC. Under normoxic conditions, the overexpression of YTHDF1 increases the translation of m^6^A-modified transcripts and induces the proliferation of NSCLC cells. However, under conditions of hypoxia or chemotherapy-induced ROS accumulation, low expression of YTHDF1 in NSCLC can reduce the translation of keap, promote the upregulation of Nrf2 and its downstream antioxidant AKR1C1, and induce cisplatin resistance [215]. Hypoxia can induce the SUMOylation of YTHDF2 in vivo and in vitro at the Lys571 site, which is repressed by oxidative stress and SUMOylation inhibitors. The SUMOylation of YTHDF2 significantly increases its binding affinity for m^6^A-modified mRNAs, leading to the degradation of transcriptome-wide mRNAs and promoting cancer progression (Figure 2(b)) [21]. Stress granules (SGs) are dynamic structure in which translationally stalled mRNAs are deposited [216–218]. Oxidative stress induces the METTL3/METTL14/WTAP-mediated deposition of m^6^A on the 5′-UTR of SGs. The m^6^A reader protein YTHDF3 has been documented to triage m^6^A-modified mRNAs to SGs under oxidative stress in HEK293 and osteosarcoma U2OS cells [219].

Oxidative stress can alter the m^6^A level by affecting the expression and activity of m^6^A enzymes and, then, determinate cell fate and physiological functions. This regulation may be an adaptive response to harmful injury, suggesting the potential role of m^6^A in stress response. A variety of epigenetic mechanisms play regulatory roles in genes expression involved in stress stimulus response. Posttranslational modifications (PTMs) of histones are produced by nonenzymatic and enzymatic processes and produce a marked effect in controlling chromatin structure and gene expression. Histone H3 trimethylation at lys36 (H3K36me3) is a transcription elongating marker that can adjust m^6^A deposition at an overall level. There is evidence that the level of H3K36me3 is positively correlated with the m^6^A level. Further studies found that the core region of H3K36me3 can be recognized and bind by METTL14, which act as a gene regulation signal to promote the interaction of m^6^A methyltransferase complex and RNA polymerase II and then enhance on m^6^A deposition on nascent RNAs, suggesting that m^6^A RNA methylation is regulated by histone modification [220]. The results partially revealed the mechanism of m^6^A-specific deposition in the transcriptome. Free radicals as regulators can regulate histone PTMs in directly and indirectly manners and participate in the epigenetic landscape [221], implying that that oxidative stress may regulate m^6^A methylation by affecting the epigenetic regulations. Overall, the achievements above confirmed the crosslink between m^6^A and oxidative stress; however, its potential mechanism remains unclear and needs further research.

### 5.2. m^6^A Modifications Affect Oxidative Stress

m^6^A, as the most common form of RNA epigenetic transcription regulation, participates in a variety of biological processes, involving oxidative stress, by affecting RNA alternative splicing, stability, translation, and subcellular localization. m^6^A RNA modification can control redox homeostasis by regulating the production of ROS, altering antioxidant genes expression, or affecting oxidative stress-related signal pathways.

#### 5.2.1. ROS Production

Mitochondria is the main source of ROS in cells [222]. m^6^A can affect the redox balance of cells by directly modifying the pathway of ROS production. The microprocessor complex, composed of Type III RNase DROSHA and RNA-binding protein DGCR8, involves in the processing of primary microRNAs (pri-miRNAs). m^6^A writer METTL3 has been proven to increase the m^6^A level of pri-miRNAs and promote their recognition and processing by DGCR8, participating in the first step of miRNA biogenesis [223, 224]. The RALY heterogeneous nuclear ribonucleoprotein (RALY, also known as hnRNPCL2) is a novel RNA-binding protein that is an important regulatory component of the DROSHA complex. It regulates the expression of mitochondrial-related ETC genes by promoting the posttranscriptional modification of specific miRNA subsets and then reprograms the mitochondrial metabolism in cancer cells. METTL3-dependent m^6^A modification is necessary for RALY-mediated miRNA maturation. METTL3 enhances m^6^A methylation in the terminal loop of pri-mir483, pri-mir877, and pri-mir676, so as to increase their interaction with the RALY complex and promote miRNA processing. The deficiency of METTL3 resulted in a significant decrease in the levels of these three pri-miRNAs and significantly affected the growth and progress of colorectal cancer. Further studies showed that METTL3 depletion induces the expression of mitochondrion-related ETC genes ATP synthase membrane subunit e (ATP5) I, ATP5G1, ATP5G3, and cytochrome c1 (CYC1) at the protein and RNA levels led to the promotion of mitochondrial respiration and accumulation of ROS. The overexpression of miR-877 can partially reverse the effect caused by METTL3 gene knockdown, indicating that METTL3-mediated m^6^A methylation of pri-miRNAs influences the mitochondrial metabolism and cell fate of colorectal cancer cells (Figure 2(c)). The overexpression of miR-877 can partially reverse the effect caused by METTL3 gene knockdown, indicating that METTL3-mediated m^6^A methylation of pri-miRNAs influence the mitochondrial metabolism and cell fate of colorectal cancer cells (Figure 2(c)) [225]. Tamoxifen has been widely used in the treatment of patients with estrogen receptor-positive breast cancer, but the effect of drug resistance has affected the patient's benefit. Adenylate kinase 4 (AK4) is a key enzyme located in the mitochondrial matrix and involved in the regulation of cell energy metabolism. In Tamoxifen-resistant breast cancer cells, the methylation level of m^6^A-specific motifs in AK4 increased significantly. Methyltransferase METTL3 can enhance AK4 expression, increase the level of ROS in breast cancer cells and activate p38 Kinase, and promote the resistance of breast cancer to Tamoxifen, and the role of demethylation ALKBH5 is opposite to that of METTL3 [226]. FTO is also involved in the oxidative stress response in clear cell renal cell carcinoma by acting as an m^6^A easer. FTO induces oxidative stress by reducing m^6^A levels of peroxisome proliferator-activated receptor *γ* coactivator-1*α* (PGC-1*α*) mRNA, a major regulator of mitochondrial metabolism, which leads to increased stability and translation of PGC-1*α* mRNA. The elevated expression of PGC-1*α* restores mitochondrial activity, induces ROS production, and suppresses tumor growth [227]. In the hepatic ischemia-reperfusion injury model, FTO plays a protective role by demethylating dynamic-related protein 1 (Drp1) mRNA and inhibits Drp1-mediated oxidative stress and mitochondrial fragmentation in the liver [228].

#### 5.2.2. Expression of Antioxidant Genes

m^6^A RNA methylation can affect the expression of antioxidant-related genes, thus regulating oxidative stress. As mentioned above, METTL3 interacts with the RNA-binding protein DGCR8 and may participate in regulating DGCR8 recognition and binding primary miRNAs, thereby influencing the maturation of miRNAs [223]. Experiments showed that the maturation of miR-873-5p modulated in a METTL3-dependent manner could activate the Nrf2 antioxidant pathway and inhibit its negative regulator Keap1 to resist colistin-induced oxidative stress and apoptosis [20]. In human HeLa cells and colon carcinoma HCT116 cells, METTL3/METTL14 catalyzes m^6^A methylation in the 3′-UTR of p21 and enhances p21 expression, leading to elevated expression of Nrf2 and inducing cell senescence under ROS stress (Figure 2(d))[229]. Other studies have shown that the endocrine-disrupting chemical di-(2-ethylhexyl) phthalate (DEHP) can increase the overall level of m^6^A methylation and increase the m^6^A modification level of Nrf2 transcripts by altering the expression of FTO and YTHDC2, thereby inhibiting the Nrf2-mediated antioxidant pathway and inducing oxidative stress [230].

In a study of human hepatoma HepG2 cells treated with different concentrations of fumonisinB_1_ (FB_1_, a common carcinogen), it was investigated whether there is cross-talk between FB_1_-induced oxidative stress and m^6^A. The results showed that FB_1_ induced intracellular ROS accumulation, and the increase in the m^6^A level was accompanied by increases in both m^6^A writers, including METLL3 and METLL14, and readers, including YTHDF1, YTHDF2, YTHDF3, and YTHDC2, and decreases in FTO and ALKBH5. FB_1_-induced m^6^A RNA methylation results in a decrease in Keap1 expression and an increase in Nrf2 expression [18]. System XC(-), a cystine/glutamate transporter, promotes cystine import and participates in GSH synthesis in response to oxidative stress [231]. The activity of System XC(-) can be regulated by SLC7A11 [232]. A study has found that the antitumor mechanism of YTHDC2 is related to the impairment of antioxidant function caused by inhibition of system XC(-) function in lung adenocarcinoma. The mechanism depends on m^6^A-mediated SLC7A11 mRNA decay, which leads to the inhibition of the downstream antioxidant process by reducing cystine uptake [233]. MALAT1 is a long noncoding RNA that has been shown to be involved in oxidative stress. Antagonism of MALAT1 can lead to transcriptional activation of Keap1 and reduction of Nrf1/2, and they mediated antioxidant gene expression and ROS accumulation [234]. A study showed that MALAT1 level was positively correlated with m^6^A modification [209], suggesting that m^6^A modification may also be involved in the regulation of oxidative stress by a MALAT1-mediated pathway.

#### 5.2.3. Upstream of ROS Generation in Cancer

Oncogenes and tumor suppressor gene pathways such as KRAS, MYC, Bcl-2, and p53 account for the abnormal oxidative stress state of cancer. The abnormal activation of KRAS occurs in pancreatic ductal adenocarcinoma, nonsmall cell lung cancer NSCLC, colorectal cancer, and other tumors which can promote ROS generation and the metabolic reprogramming of cancer cells by regulating mitochondria function [78, 235]. A bioinformatics analysis, including 1017 NSCLC patients with copy number variation (CNV) data, has shown that the high expression of FTO is positively correlated with the activation of the KRAS signal transduction pathway [236]. Overexpression of m^6^A reader YTHDF2 inhibits the activation of MEK and ERK which are downstream of Ras and impairs hepatocellular carcinoma progression [237]. Moreover, B-Raf protooncogene (BRAF) and MEK inhibitors can remodel mRNA translation in melanoma which is related to m^6^A level, suggesting that m^6^A affects tumor growth and drug resistance by participating in Ras signal regulation [238].

Expression of endogenous oncogenic alleles of MYC can increase Nrf2 transcription and its mediated antioxidant program to detoxify intracellular ROS [90]. Another study has shown that MYC oncoprotein increases ROS produced by mitochondrial ETC [77]. These results suggest that MYC plays a complex role in oxidative stress through different mechanisms. m^6^A can directly modify MYC mRNA. A research exhibits that MYC act as a direct target of m^6^A in haematopoietic stem cells by RNA sequencing [239]. The increase of m^6^A modifications of MYC mRNA facilitates the binding of YTHDF1, then promotes MYC expression and subsequent metabolic reprogramming and proliferation in lung adenocarcinoma [240]. YTHDF2 can stabilize MYC transcripts to mediate the viability of glioblastoma stem cells in an m^6^A-dependent manner [241]. Similarly, IGF2BP1 recognizes m^6^A modification of MYC mRNA to increase the stability and expression MYC, thereby promoting tumorigenesis [242]. In addition to directly affecting the transcription and translation of MYC, m^6^A can also regulate MYC expression by lncRNA. Lung Cancer Associated Transcript 3 (LCAT3) is a newly identified oncogenic lncRNA, which is upregulated in lung adenocarcinoma through METTL3 mediated m^6^A methylation. LCAT3 can activate the transcription of MYC and promote the survival and progression of lung adenocarcinoma [243].

Bcl-2 is one of the most important oncogenes in the field of apoptosis which can maintain mild a prooxidant state by enhancing mitochondrial respiration to sustain the survival of cancer cells [79]. Single-nucleotide-resolution mapping combining ribosome profiling shows that the translation of Bcl-2 mRNAs is governed by m^6^A methylation in acute myeloid leukemia cells [244]. Other studies have also demonstrated that Bcl-2 expression is regulated by m^6^A-dependent pathways in NSCLC [245] and breast cancer [246].

p53 is an important tumor suppressor gene participating in cell cycle arrest, apoptosis, and senescence. As a crucial transcription factor, wild-type p53 plays a coordinating role in oxidative stress. p53 showed antioxidant capacity in response to low levels of oxidative stress to clear ROS and maintain cell survival; in the face of high levels of oxidative stress, p53 can promote ROS production and further induce cell death [247, 248]. p53^R273H^ is the missense mutation form of p53. It has been observed that there is m^6^A methylation at the 273 mutated codons (G to A) of p53 pre-mRNA which can increase the splicing of pre-mRNA and, then, promote the expression of p53^R273H^ protein. Subsequent studies confirmed this process is mediated by METTL3. Upregulation of METTL3 can promote the expression of p53^R273H^ and mediate the drug resistance of colon cancer cells [249]. A study uses 1 *μ*M arsenite to induce an overall increase in m^6^A RNA modifications showed that decreased activity, phosphorylation, acetylation, and nuclear expression levels of p53 in human keratinocyte HaCaT cells with elevated m^6^A levels. METTL3 knockout confirmed that m^6^A modification influences the expression and activity of p53 by downregulating positive regulatory factors and upregulating negative regulatory factors of p53 [250]. p21 is a cyclin-dependent kinases inhibitor downstream of p53. 3′UTR of p21 occurs the combined modification of NOP2/Sun RNA methyltransferase 2- (NSUN2-) mediated 5-methylcytosine (m^5^C) and METTL3/METTL4-mediated m^6^A methylation. They synergistically enhance the expression of p21 at the translation level in response of oxidative stress-induced cellular senescence [229].

m^6^A modification alters the redox state by regulating ROS production, governing antioxidant gene expression, and affecting oncogene signals that induce oxidative stress. In general, these findings suggest that the mutual regulation does occur between oxidative stress regulators and m^6^A modifications (Tables 4 and 3). However, the interplay of m^6^A modification and oxidative stress is complex and dynamic, and its regulatory mechanism needs to be systematically and deeply studied.

### 5.3. Role of Oxidative Stress and m^6^A Modification in Cancer Therapy

Conventional anticancer therapies, such as radiotherapy and chemotherapy, are closely related to ROS generation. Cytotoxic drugs and ionizing radiation can cause DNA damage and apoptosis mediated by ROS. Therefore, the regulation of oxidative stress plays an important role in cancer therapy. As mentioned earlier, elevated ROS levels induce tumor growth and development, so antioxidant supplementation may be a potential therapeutic strategy for ROS-induced cancer. Vitamin C is one of the most common antioxidants. In multiple myeloma [251], pancreatic cancer [252], and ovarian cancer [253], vitamin C can function either in cooperation with chemotherapy or alone to play an anticancer role. The role of vitamin C in the anticancer immune response has been studied. In preclinical models, a high dose of vitamin C can regulate immune cell infiltration in the tumor microenvironment, enhance the cytotoxicity of adoptively transferred T cells, and cooperate with immune checkpoint blockers to treat a variety of cancers [254]. Vitamin E is also considered to be an effective antioxidant that plays a role in reducing the risk of hepatocellular carcinoma [255], prostate cancer [256], and breast cancer [257]. Nrf2 is a major regulator of the tumor antioxidant response [33]. Many natural and synthetic compounds, including curcumin, resveratrol, sulforaphane, and RTA 405, are considered to be Nrf2 activators and participate in the chemoprevention of cancer [258].

It is worth noting that some studies have shown that antioxidant treatment may increase the risk of certain cancers and promote their progression. For example, the antioxidants vitamin E and N-acetylcysteine can improve oxidative damage in the lung tissue of mice but may induce the development and progression of lung cancer [259, 260]. A large-sample, multicenter, prospective cohort study that explored the role of vitamin E in prostate cancer prevention found that the long-term use of supplemental vitamin E may increase the risk of prostate cancer in healthy men [261]. These results suggest that some types of cancer may survive under the protective effects of antioxidants and that prooxidants may be effective in such cases. Common chemotherapy drugs, including paclitaxel, platinum complex, adriamycin, and antimetabolites, can cause oxidative stress. Arsenic trioxide can induce the apoptosis of many kinds of cancer cells by increasing ROS levels [262]. The cytotoxicity of 5-fluorouracil is related to the production of ROS. Cancer cells are resistant to 5-fluorouracil through antioxidant mechanisms [263]. ROS also mediates oxidative damage and cell death induced by ionizing radiation [264]. In addition, the anticancer effects of many drugs directly targeting ROS metabolic pathways have been confirmed. These drugs fall into two categories [265]. The first is drugs that target glutathione; for example, NOV-002 is a kind of oxidized glutathione that can inhibit DNA repair and play a direct anticancer role. In addition, it can be used as a substrate to participate in s-glutathionation and affect a variety of signaling pathways. Clinical data show that the combination of NOV-002 and cisplatin can increase the survival of patients with NSCLC and increase the tolerance of patients to chemotherapy [266]. The second kind of drug targets thioredoxin, including auranofin, PX-12, and dimesna. PX-12 is an irreversible inhibitor of thioredoxin-1 that can inhibit cell growth, increase ROS levels, and induce apoptosis in acute lymphoblastic leukemia [267], gastric cancer, and hepatocellular carcinoma [268]. At present, some redox regulatory drugs have entered the clinical trial stage and are expected to become candidates for cancer treatment [269].

Some agents targeting oxidative stress may play a role in regulating m^6^A modifications. Sulforaphane (SFN) is not only an oxidative stress regulator but also an epigenetic modulator. On the one hand, SFN plays an antioxidation role by activating Nrf2; on the other hand, it can mediate the depletion of GSH and increase the production of ROS [270]. Studies have found that the effect of SFN is concentration dependent. Using low-dose SFN to treat breast cancer cell lines can reduce the overall m^6^A level, and induce oxidative stress, G2/M cell cycle arrest, and apoptosis which benefit to cancer inhibition [271]. Epigallocatechin gallate (EGCG) is a kind of green tea polyphenols which play a role in antioxidation [272]. EGCG treated adipocytes shown a decrease of FTO expression and increase of total m^6^A-methylated RNA levels resulting in inhibition of adipocyte differentiation [273]. Both resveratrol and curcumin are natural antioxidants which have been considered as effective agents for cancer chemoprevention [274]. The combination of them can lead to the downregulation of the level of m^6^A [275]. Another study had shown that resveratrol can reducing the ROS accumulation induced by mycotoxin Aflatoxin B1 thereby decreasing m^6^A-modified RNA levels [276]. These results exhibit the association between oxidative stress and m^6^A modification. The extensive and profound effect of ROS on the level of m^6^A implies that m^6^A modification may play a role in the effects of many anticancer drugs related to oxidative stress. On the other side, using oxidative modulators to affect m^6^A modification of specific transcripts may be a potential cancer treatment. m^6^A enzymes and levels may become biomarkers of anticancer therapy. The discovery and development of new drugs targeting oxidative stress is a very promising research direction.

## 6. Conclusions and Future Perspectives

One feature that distinguishes cancer cells from normal cells is their aberrant oxidation and antioxidant system. The study of the mechanisms of oxidative stress in cancer may give rise to a new field of redox medicine in which oxidants and antioxidants may become effective treatment strategies for cancer. However, due to the extensive and complex effects of ROS in cancer, their roles as signaling molecules cannot be simply defined. Increasing evidence demonstrates that oxidative stress has the ability to regulate m^6^A RNA methylation, which may be correlated with the degree of ROS accumulation and can affect tumor progression. Similarly, m^6^A methylation has proven to affect the biological functions of cancer cells, including growth, progression, senescence, and apoptosis, by affecting ROS levels. In this review, we summarize the dual functions and effects of oxidative stress in cancer cells and the general mechanisms underlying its effects. The changes in m^6^A methylation levels in response of oxidative stress and the regulation of intracellular ROS levels via m^6^A modification are emphasized. These findings not only provide new clues for elucidating the mechanisms of different cell responses to oxidative stress but also give rise to new prospects for targeting m^6^A modification pathways to regulate oxidative stress.

## Figures and Tables

**Table 1:** Roles of m^6^A in cancer.

m^6^A enzyme	Cancer type	Role	Change in target RNA	Function of m^6^A enzyme	References
METTL3	Acute myeloid leukemia	Oncogene	MYC↑, BCL2↑, PTEN↑	Inhibiting cell differentiation and apoptosis, increasing cell growth	[244]
	Breast cancer	Oncogene	HBXIP↑	Promoting cell proliferation, inhibiting apoptosis	[246, 277]
	Bladder cancer	Oncogene	miR-221/222↑, PTEN↓	Promoting cell growth	[278]
		Oncogene	SETD7↓, KLF4↓	Promoting cell proliferation and metastasis	[279]
	Colorectal cancer	Oncogene	SOX2↓	Promoting tumorigenesis and cell metastasis	[280]
		Tumor suppressor	p-p38↓, p-ERK↓	Inhibiting cell proliferation, migration, and invasion	[281]
	Glioblastoma	Oncogene	SRSFs↑	Promoting tumor growth and progression	[282]
		Oncogene	ADAR1↑	Promoting cancer progression	[283]
	Lung cancer	Oncogene	JUNB↑	Increasing TGF-*β*-induced EMT	[284]
		Oncogene	BRD4↑	Promoting oncogenic transformation and tumor growth	[285]
	Liver cancer	Oncogene	SOCS2↓	Promoting cell proliferation, migration, and colony formation	[286]
		Oncogene	RDM1↓	Increasing cell proliferation	[287]
METTL3, METTL14	Endometrial cancer	Tumor suppressor	PHLPP2↑, mTORC2↓	Inhibiting cell proliferation	[288]
	Glioblastoma	Tumor suppressor	ADAM19↑, EPHA3↑, KLF4↑, CDKN2A↓, BRCA2↓, TP53I11↓	Suppressing tumor genesis, growth, and self-renewal	[289]
METTL14	Acute myeloid leukemia	Oncogene	MYC↑, MYB↑	Inhibiting cell differentiation, increasing cell proliferation	[290]
	Breast cancer	Oncogene	DROSHA↑	Enhancing breast cancer stem-like cell stemness maintenance	[291]
	Colorectal cancer	Tumor suppressor	SOX4↓	Inhibiting EMT	[292]
		Tumor suppressor	lncRNA XIST↓	Suppressing proliferation and metastasis	[293]
	Pancreatic cancer	Oncogene	PERP↓	Promoting tumor growth and metastasis	[294]
WTAP	Liver cancer	Oncogene	ETS1↓	Promoting cell proliferation and tumor growth	[295]
FTO	Acute myeloid leukemia	Oncogene	MYC↑, CEBPA↑	Promoting cell proliferation and viability, inhibiting cell-cycle arrest and apoptosis	[296]
		Oncogene	ASB2↓, RARA↓	Enhancing cell transformation and leukemogenesis, inhibiting cell differentiation	[297]
	Liver cancer	Oncogene	ALDOA↑	Promoting cell growth under hypoxia	[298]
	Ovarian cancer	Tumor suppressor	PDE1C↓, PDE4B↓	Inhibiting the tumor self-renewal, suppressing tumorigenesis	[299]
ALKBH5	Acute myeloid leukemia	Oncogene	TACC3↑	Promoting tumor development and self-renewal	[300]
	Lung cancer	Tumor suppressor	YAP↓	Inhibiting tumor growth and metastasis	[301]
	Glioblastoma	Oncogene	FOXM1↑	Promoting cell proliferation	[51]
	Pancreatic cancer	Tumor suppressor	PER1↑	Reducing cell proliferation, migration, and invasion, suppressing tumor growth	[302]
YTHDF1	Lung cancer	Oncogene	CDK2↑, CDK4↑, cyclind1↑, Keap1↑	Promoting cell proliferation and xenograft tumor formation	[215]
	Liver cancer	Oncogene	EGFR↑	Promoting cell viability and metastasis	[303]
	Ovarian cancer	Oncogene	EIF3C↑	Facilitating tumor genesis and metastasis	[304]
YTHDF2	Glioblastoma	Oncogene	MYC↑, VEGFA↑	Maintaining glioblastoma stem cell stemness	[241]
	Liver cancer	Tumor suppressor	IL11↓, SERPINE2↓	Reducing tumor inflammation and causing vascular abnormalities	[59]
	Prostate cancer	Oncogene	LHPP↓, NKX3-1↓	Inducing tumor proliferation and migration	[305]
YTHDF3	Breast cancer	Oncogene	ST6GALNAC5↑, GJA1↑, EGFR↑	Controlling the interaction of cancer and brain microenvironment, inducing brain metastasis	[306]
	Colorectal cancer	Oncogene	LncRNA GAS5↓	Promoting cancer progression	[307]
IGF2BP1	Colorectal cancer	Oncogene	c-Myc↑	Promoting tumorigenesis	[308]
	Ovarian, liver, and lung cancer	Oncogene	SRF↑, PDLIM7↑, FOXK1↑	Promote cell growth and invasion	[309]
IGF2BPs	Cervical and liver cancer	Oncogene	MYC↑	Promoting tumorigenesis	[310]

**Table 2:** Effects of oxidative stress in cancers.

Cancer type	Gene involved	Function of ROS	Description	References
Acute myeloid leukemia	TIGAR	Tumor suppressor	Knockdown of TIGAR promoting ROS-mediated apoptosis and antiproliferation	[311]
	NOX	Tumor promoter	Activation of NOX increases extracellular ROS level promoting the proliferation of acute myeloid leukemia blasts	[312]
Breast cancer	p53	Tumor suppressor	p53 activation induced by ROS can promote necrosis and apoptosis of cancer cells	[313]
	SOD2	Tumor promoter	Upregulation of SOD2 induces elevated ROS to sustain AMPK-activated signal to promote aerobic glycolysis and malignant transformation	[314]
	ZEB1; GPX4	Tumor promoter	ZEB1 inhibits transcription of GPX4, increases ROS accumulation and EMT, which promote breast cancer progression	[315]
Colorectal cancer	HIF-2*α*	Tumor suppressor	Activated HIF-2*α* induces ROS production by an iron-dependent pathway which led colorectal cancer cell death	[316]
	ANGPTL4; NOX4	Tumor promoter	ANGPTL4/NOX4 axis maintains the metastatic ability of colorectal cancer cells via increasing ROS, MMP1, and MMP9 levels	[317]
Glioblastoma	PRDX3	Tumor suppressor	Prohibitin maintains the stability of PRDX3 to reduce the production of ROS, maintain glioblastoma stemness and promote the resistance of gliomas stem-like cells to radiotherapy	[318]
	TRAP1; SIRT3	Tumor suppressor	TRAP1 cooperate with SIRT3 to reduce ROS production and promotes stress adaptation of glioblastoma cancer stem cells	[319]
Gastric cancer	NNT	Tumor suppressor	NNT deficiency can significantly reduce NADPH and significantly induce ROS production and apoptosis under stress.	[320]
	GRIM-19; Nrf2	Tumor promoter	GRIM-19 deficiency accelerates gastric cancer metastasis via abnormal oxidative stress and ROS-driven Nrf2 activation	[321]
Hepatocellular carcinoma	UBQLN1; PGC1*β*	Tumor suppressor	Elevated expression of UBQLN1 induces PGC1*β* degradation to promote sorafenib resistance of hepatocellular carcinoma cells by reducing mitochondrial ROS production	[322]
	PKC*λ*/*ι*; Nrf2	Tumor promoter	Loss of PKC*λ*/*ι* induces ROS generation promoting hepatocellular carcinoma in a Nrf2-dependent manner	[323]
Lung cancer	IL-15; mTOR	Tumor promoter	NK cells activate thioredoxin system through IL-15/mTOR axis to adapt to high ROS level in tumor microenvironment	[324]
	AK4; HIF-1*α*	Tumor promoter	Upregulation of AK4 enhances expression of HIF-1*α* through increasing ROS production, and then EMT was induced in hypoxia condition	[325]
	AIM2; MAPK/ERK; MFN2	Tumor promoter	Knockdown of AIM2 upregulates MFN2 and enhances the mitochondrial fusion, resulting in the reduction of mitochondrial ROS production, which in turn induces the inactivation of the MAPK/ERK pathway and hinders the progress of non-small cell lung cancer	[326]
	Nestin; Keap1; Nrf2;	Tumor suppressor	Nestin competed with Nrf2 for binding to Keap1, leading to Nrf2 escape and downstream antioxidant gene expression, which promotes the resistance of NSCLC to oxidative stress	[327]
Melanoma	ANGPT2	Tumor suppressor	Silence of Angpt2 expression significantly increases the level of intracellular ROS and activation of downstream MAPK pathway, thus resulting in the metastatic colonization of melanoma	[328]
	Akt	Tumor promoter	Akt overexpression can induce the expression of NOX4, increase the level of ROS, increase the expression of VEGF, increase angiogenesis, and promote the aerobic glycolysis of melanoma cells	[329]
Ovarian cancer	RAD51	Tumor promoter	Loss of RAD51 accelerates mitochondrial ROS accumulation and DNA damage which can be weakened by treatment of antioxidant N-acetylcysteine	[330]
Pancreas cancer	UCP2; Akt; mTOR	Tumor suppressor	Inhibition of UCP2 plays an anticarcinogenic role in pancreatic adenocarcinoma cells via activating ROS/Akt/mTOR axis	[331]
Renal cell carcinoma	TAZ; EMP1; NOX4;	Tumor suppressor	Nuclear translocation of TAZ upregulates EMP1 expression, thereby increasing the mRNA level of NOX4 and inducing ferroptosis of renal cell carcinoma cells via elevated lipid ROS	[332]

**Table 3:** m^6^A RNA methylation regulates oxidative stress in cancer.

m^6^A enzyme	Change of m^6^A modification	ROS levels	Cancer type	Mechanism	Biofunction in cancer	References
METTL3; ALKBH5	m^6^A level↑	↑	Breast cancer	METTL3 enhances AK4 expression, increase the level of ROS in breast cancer cells and activate p38 Kinase	Promoting the resistance of breast cancer to Tamoxifen	[226]
METTL3	Unknown	↑	Colorectal cancer	METTL3 facilitates the processing of miR-483, miR-676, and miR-877 which regulating the expression of mitochondrial related ETC genes	Promoting cancer growth and progression	[225]
METTL3; METTL14	Unknown	↓	Colon carcinoma	METTL3/METTL14 catalyzes the m^6^A methylation of p21 and enhances p21 expression leading to elevated expression of Nrf2	Inducing cell senescence	[208, 229]
FTO	Unknown	↑	Clear cell renal cell carcinoma	FTO increases stability and translation of PGC-1*α* mRNA thereby resulting in oxidative stress	Inducing ROS production and suppressing tumor growth	[227]
YTHDF1	YTHDF1↓	↓	Nonsmall cell lung cancer	Knockdown of YTHDF1 reduces the translation of keap, upregulates Nrf2 and its downstream antioxidant in response of cisplatin-induced ROS	Adapting to oxidative stress; Inducing cisplatin resistance in nonsmall cell lung cancer	[215]
METTL3; YTHDF2	METTL3↑ SUMOylation of YTHDF2	↑	Lung adenocarcinoma	YTHDF2 can be SUMOylated at K571 in hypoxia or oxidative stress condition	Promoting mRNA degradation and cancer progression	[21]
YTHDC2	Unknown	↑	Lung adenocarcinoma	YTHDC2 regulates SLC7A11 mRNA decay, which leads to the inhibition system XC(-) function, thus impairing the antioxidant function	Inhibiting tumorigenesis	[233]

**Table 4:** ROS regulate m^6^A modification.

Oxidative stress activators	Cell type	Change of m^6^A components	Biofunction	References
Low dose of NaAsO_2_	Human keratinous HaCaT cells	METTL3↑; METTL14↑; WTAP↑; FTO↓	Moderate level of ROS-facilitating cell survival via elevated m^6^A levels in HaCaT cells	[208]
High dose of NaAsO_2_	Human keratinous HaCaT cells	METTL3↓; METTL14↓;WTAP↓;FTO↑	High level of ROS inducing cell death by decreased m^6^A levels in HaCaT cells	[208]
Hypoxia	Breast cancer stem cells	ALKBH5↑	Decreasing NANOG mRNA methylation, enhancing the expression of NANOG transcripts, and inducing breast cancer stem cell phenotype	[212]
H_2_O_2_	Hematopoietic stem/progenitor cells	ALKBH5 m^6^A demethylase activity↓	Participating in DNA damage repair and protecting genomic integrity of cells	[213]
Bmal1 deletion	Hepatic cells	METTL3↑; YTHDF2↑	Increasing PPaR*α* m^6^A abundance, decreasing its expression, and promoting lipid accumulation	[214]
Hypoxia	NSCLC cells	YTHDF1↑	Playing a role in hypoxia adaptation of NSCLC through Keap1-Nrf2-AKR1C1 axis	[215]
Hypoxia	Lung adenocarcinoma cells	YTHDF2 SUMOylation at the Lys571 site	Promoting degradation of transcriptome-wide mRNAs and cancer progression	[21]

## Data Availability

No data were used to support this study

## Abbreviations

ADAM19: ADAM metallopeptidase domain 19
ADAR1: Adenosine deaminase RNA specific
AIM2: Absent in melanoma 2
ALDOA: AldolaseA
AMPK: AMP-activated protein kinase
ANGPT2: Angiopoietin 2
ANGPTL4: Angiopoietin-like 4
ASB2: Ankyrin repeat and SOCS box containing 2
BRCA2: BRCA2 DNA repair associated
BRD4: Bromodomain-containing protein 4
CDK: Cyclin-dependent kinase
CDKN2A: Cyclin-dependent kinase inhibitor 2A
CEBPA: CCAAT enhancer-binding protein alpha
DROSHA: Drosha ribonuclease III
EGFR: Epidermal growth factor receptor
EIF3C: Eukaryotic translation initiation factor 3 subunit C
EPHA3: EPH receptor A3
ETS1: ETS protooncogene 1
FOXM1: Forkhead box M1
GAS5: Growth arrest-specific 5
GJA1: Gap junction protein alpha 1
GRIM-19: Gene associated with retinoic and interferon-induced mortality 19 protein
HBXIP: Hepatitis B X-interacting protein
IL11: Interleukin 11
JUNB: JunB protooncogene
KLF4: Kruppel like factor 4
LHPP: Phospholysine phosphohistidine inorganic pyrophosphate phosphatase
LncRNA: Long noncoding RNA
MALAT1: Metastasis-associated lung adenocarcinoma transcript 1
MFN2: Mitofusin 2
mTOR: Mechanistic target of rapamycin kinase
MYB: MYB protooncogene
MYC: MYC protooncogene
NKX3-1: NK3 homeobox 1
NNT: Nicotinamide nucleotide transhydrogenase
PDE: Phosphodiesterase
PER1: Period circadian regulator 1
PERP: p53 apoptosis effector related to PMP22
PGC1*β*: Peroxisome proliferator-activated receptor *γ* coactivator 1*β*
PHLPP2: PH domain and leucine-rich repeat protein phosphatase 2
PKC*λ*/*ι*: Protein kinase C *λ*/*ι*
PRDX3: Peroxiredoxin3
RAD51: RAD51 recombinase
RARA: Retinoic acid receptor alpha
RDM1: RAD52 motif 1
SERPINE2: Serpin family E member 2
SETD7: SET domain containing 7
SIRT3: Sirtuin3
SOCS2: Suppressor of cytokine signaling 2
SOX2: SRY (sex determining region Y)-box 2
SRSFs: serine- and arginine-rich splicing factors
ST6GALNAC5: ST6 N-acetylgalactosaminide alpha-2,6-sialyltransferase 5
TACC3: Transforming acidic coiled-coil containing protein 3
TP53I11: Tumor protein p53 inducible protein 11
TRAP1: Tumor necrosis factor-receptor-associated protein 1
UBQLN1: Ubiquilin 1
UCP2: Mitochondrial uncoupling protein 2
UTRs: Untranslated regions
VEGF: Vascular endothelial growth factor
VEGFA: Vascular endothelial growth factor A
VHL: Von Hippel-Lindau
XIST: X inactive-specific transcript
YAP: Yes1 associated transcriptional regulator
ZEB1: Zinc finger E-box binding homeobox 1.
